# Label-Free Quantitative Proteomics Analysis of COVID-19 Vaccines by Nano LC-HRMS

**DOI:** 10.3390/vaccines12091055

**Published:** 2024-09-15

**Authors:** Hengzhi Zhao, Wendong Li, Jingjing Liu, Xiao Li, Hong Ji, Mo Hu, Min Li

**Affiliations:** 1 NMPA Key Laboratory for Safety Research and Evaluation of Innovative Drugs, Beijing Key Laboratory of Analysis and Evaluation on Chinese Medicine, Beijing Institute for Drug Control, Beijing 102206, China; 2 Changping Laboratory, Beijing 102206, China

**Keywords:** vaccine, proteomics, nano LC-HRMS, COVID-19, host cell proteins

## Abstract

A nanoliter liquid chromatography–high resolution mass spectrometry-based method was developed for quantitative proteomics analysis of COVID-19 vaccines. It can be used for simultaneous qualitative and quantitative analysis of target proteins and host cell proteins (HCPs) in vaccine samples. This approach can directly provide protein information at the molecular level. Based on this, the proteomes of 15 batches of COVID-19 inactivated vaccine samples from two companies and 12 batches of COVID-19 recombinant protein vaccine samples from one company were successfully analyzed, which provided a significant amount of valuable information. Samples produced in different batches or by different companies can be systematically contrasted in this way, offering powerful supplements for existing quality standards. This strategy paves the way for profiling proteomics in complex samples and provides a novel perspective on the quality evaluation of bio-macromolecular drugs.

## 1. Introduction

In 2019, the COVID-19 virus ravaged the world, and the outbreak of the pandemic affected every aspect of people’s lives [1]. As a great invention, vaccines are important in preventing the transmission of viruses and reducing the risk of infection [2]. There were several kinds of vaccines that dominated during the epidemic, such as inactivated vaccines, recombinant vaccines and mRNA vaccines [3,4]. Inactivated vaccines, due to their high safety and mature production technology, comprised the highest number of vaccinations during the COVID-19 epidemic. Among these kinds, inactivated vaccines and recombinant vaccines both stimulate human bodies to produce antiviral antibodies by immune stimulation through the target proteins of the virus; in the case of COVID-19, this is the S proteins [5]. Thus, the determination of the S protein is of great significance for the quality control of vaccine products. Meanwhile, the residual of host cell proteins (HCPs) is inevitably introduced in vaccine production process. These HCPs can cause unknown immune responses which may induce serious toxicity and side effects to immune recipients [6]. Therefore, it is necessary to determine HCP residues in such vaccine products.

Generally, the detection of S proteins and HCP residues relies on enzyme-linked immunosorbent assay (ELISA)-based assays [7]. However, there are still some limitations in the measurement. Firstly, for target protein analysis, this method can only make a general quantitation which cannot provide additional subtype information of the target proteins at the molecular level. Second, the absolute quantification of S proteins by ELISA-based methods needs to rely on the usage of target protein standards. For the quality control of vaccine production in industry, quantitative control between batches is usually conducted according to a specific batch of reference product; thus, it is difficult to achieve comparisons between different companies’ products. In addition, the ELISA-based methods cannot cover all kinds of HCPs, resulting in the underestimate of HCP content [8,9]. Moreover, the ELISA-based methods cannot simultaneously analyze the content of S proteins and HCPs at the same time, which increases the procedures and duration of detection.

Liquid chromatography–mass spectrometry (LC-MS) is an analytical technology with high selectivity, high sensitivity and high-throughput features [10,11,12]. Since corresponding antibodies are not required, LC-MS has the advantage of rapid method development and is therefore more suitable for emergency situations such as pandemics, avoiding risks such as poor antibody specificity. The advancement of LC-MS in proteomics enables the accurate qualitative and quantitative analysis of proteins, especially in complex samples [13,14,15,16]. Based on the analysis capacity of LC-MS at the molecular level, it is very suitable for the profile of related proteins in bio-macromolecular samples such as vaccines. Due to its universal capacity, the LC-MS method can be used for global monitoring during production (e.g., virus/host cell metabolism, MOI optimization, etc.). Not only the same vaccines produced in different batches or by different companies, but also the vaccines of different pathogens and the different kinds of vaccines (inactivated, recombinant and so on) can be analyzed in one set of methods.

Herein, we developed a nano LC-MS method for the simultaneous analysis of S proteins and HCP residues of COVID-19 inactivated vaccine products and recombinant vaccine products. Based on this, the content of S proteins and HCP residue could be quantified according to the spiked standard proteins. Additionally, different batches, different companies and different kinds of vaccine products were successfully compared, which may be valuable to improving the manufacturing technique of vaccines in the future.

## 2. Materials and Methods

### 2.1. Vaccine Samples and Standard Substance

Each of three batches of COVID-19 (wild-type strain) inactivated vaccine final bulk from two companies (company A and B) were marked with AW1, AW2, AW3, BW1, BW2 and BW3, respectively. Three batches of COVID-19 (Gamma strain) inactivated vaccine final bulk from company A were marked with AG1, AG2 and AG3, respectively. Each of three batches of COVID-19 (Omicron strain) inactivated vaccine final bulk from two companies (company A and B) were marked with AO1, AO2, AO3, BO1, BO2 and BO3, respectively. Each of three batches of COVID-19 recombinant protein vaccine final bulk from company C were marked with CW1, CW2, CW3, CB1, CB2, CB3, CD1, CD2, CD3, CO1, CO2 and CO3, respectively (W represents wild-type strain, B represents Beta strain, D represents Delta strain and O represents Omicron strain). One batch of hepatitis A virus inactivated vaccine final bulk from company F was marked with F. One batch of Haemophilus infiuenzae type b conjugate vaccine final bulk from company G was marked with G. Pierce intact protein standard mix was purchased from ThermoFisher Scientific (Waltham, MA, USA).

### 2.2. Reagents and Materials

Dithiothreitol (DTT) and 2-iodoacetamide (IAA) were bought from Sigma-Aldrich (St. Louis, MO, USA). Ammonium bicarbonate was bought from Acros Organics (Fair Lawn, NJ, USA). RapiGest SF and trypsin were purchased from Waters Corporation (Milford, MA, USA) and Promega (Madison, WI, USA), respectively. Formic acid (LC-MS grade) and acetonitrile (LC-MS grade) were respectively bought from ThermoFisher Scientific (Waltham, MA, USA) and Merck (Darmstadt, Germany). A 2019-nCoV Spike protein ELISA kit was bought from Sino Biological (Beijing, China). Ultrapure water (18.2 MΩ·cm) was purified through a Millipore Milli Q water purification system and was used in all experiments.

### 2.3. Instruments

An Easy-nLC 1200 (ThermoFisher Scientific, Waltham, MA, USA), Orbitrap Eclipse mass spectrometer (ThermoFisher Scientific, Waltham, MA, USA) and centrifuge (ThermoFisher Scientific, Waltham, MA, USA) were used in this study.

### 2.4. Enzymolysis of Vaccine Samples and Standard Substance

First, 10 µL of Pierce intact protein standard mix solution (200 μL of 50 mM NH_4_HCO_3_ buffer was used to dissolve one bottle of Pierce intact protein standard mix) and 110 µL of RapiGest SF (1 mg/mL, dissolved in 50 mM NH_4_HCO_3_ buffer) were added to a 100 µL vaccine sample. After being gently mixed, the mixture was kept at 60 °C for 15 min. Then, 10 µL of DTT (0.5 M, dissolved in 50 mM NH_4_HCO_3_ buffer) was added to the above mixture and incubated at 60 °C for another 60 min. After cooling down to room temperature, 10 µL of IAA (1 M, dissolved in 50 mM NH_4_HCO_3_ buffer) was added and kept in the dark for 30 min at room temperature. After that, all of the mixture was transferred to a 3K ultrafiltration device and centrifuged for 10 min under 21,000× *g* of centrifugal force. Then, 100 μL of NH_4_HCO_3_ buffer (50 mM) was added to run a new round of the ultrafiltration process. This process was performed twice. Subsequently, 100 μL of NH_4_HCO_3_ buffer (50 mM) and 10 μL of trypsin (0.2 mg/mL, dissolved in 50 mM NH_4_HCO_3_ buffer) were added to the upper chamber of the above ultrafiltration device and gently mixed. After enzymolysis at 37 °C overnight, 3 μL of formic acid was added, followed by centrifugation under 21,000× *g* of centrifugal force for 10 min. Finally, the filtrate was collected for LC-MS analysis.

For control experiment, initially, 10 µL of Pierce intact protein standard mix solution (200 μL of 50 mM NH_4_HCO_3_ buffer was used to dissolve one bottle of Pierce intact protein standard mix) was mixed with 110 µL of RapiGest SF (1 mg/mL, dissolved in 50 mM NH_4_HCO_3_ buffer). The following process was the same as for the vaccine samples.

### 2.5. Chromatography

An Acclaim PepMap^TM^ 100 C18 (75 μm × 20 mm, 2 µm) (ThermoFisher Scientific, Waltham, MA, USA) was used as a trap column and an Acclaim PepMap^TM^ 100 C18 (75 μm × 250 mm, 2 µm) (ThermoFisher Scientific, Waltham, MA, USA) was used as an analytical column. For mobile phases, 0.1% formic acid in water was used as phase A and 0.1% formic acid in 80% acetonitrile as phase B. The flow rate was 0.3 μL/min. The volume of sample injection was 1 μL. The details of the eluent gradient are shown in Table 1.

### 2.6. Mass Spectrometry

Nanospray source (NSI) was used. The mode was positive ion; capillary voltage was 2.0 kV; capillary temperature was 300 °C; scan range (*m*/*z*) was 350–1800; and orbitrap resolution was 120,000 (MS), 30,000 (MS/MS).

### 2.7. LC-MS Data Analysis

Database searching was performed on Proteome Discoverer. Label free quantification was performed with the match-between-runs function. Raw protein abundances were normalized using the added six-protein standard. Differential regulated proteins were defined as more than two folds of change of abundance and a *p*-value less than 0.05 (student’s *t*-test).

## 3. Results and Discussion

### 3.1. Method Validation

#### 3.1.1. Selectivity

**Figure 1:**
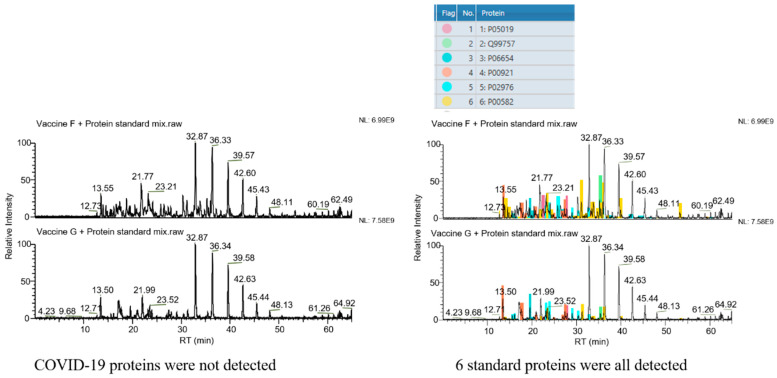
Selectivity of the method. Left column: COVID-19 structural proteins set as the target proteins. No peak belonging to COVID-19 structural proteins is seen. Right column: 6 standard proteins set as the target proteins. Peaks belonging to these 6 standard proteins are all found in spectra (the 6 colors represent the 6 standard proteins, as marked in the upper form, respectively).

The selectivity of the assay was verified by analysis of the vaccine samples F and G (non-COVID-19 vaccines). As shown in Figure 1, the target proteins could not be detected in these two vaccine samples when COVID-19 structural proteins were defined as the target proteins. However, all six protein standard substances were observed in the result if standard proteins were set as the targets. These results demonstrated the good selectivity of the assay and its potential in diverse protein analysis.

#### 3.1.2. Repeatability

**Figure 2:**
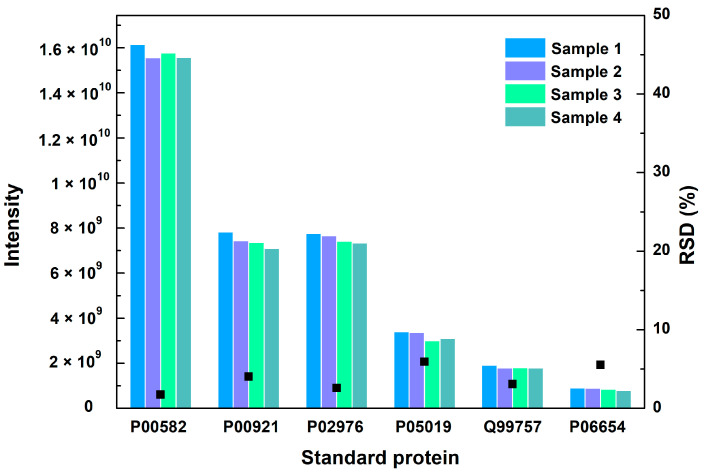
The MS intensity and their RSD values of 6 standard proteins in 4 vaccine samples. Black square represents the RSD value.

The repeatability of the method was checked by measuring the relative standard deviation (RSD) of six standard proteins in different vaccine samples. The RSD values of the six standard proteins were 1.7%, 4.1%, 2.6%, 5.9%, 3.1% and 5.5%, respectively, which shows no significant difference in four samples (Figure 2). The repeatability of the method met the requirements for analysis.

#### 3.1.3. Accuracy

The RSD values in six independent injections were calculated as 7.71%, 7.66%, 8.66%, 5.14%, 6.14% and 8.23% based on the six different standard proteins, respectively, indicating a good accuracy of sample injection.

#### 3.1.4. Sensitivity

The concentration of minimum abundance of the proteins was calculated to be 1 pM according to internal standard proteins.

### 3.2. Quantification of COVID-19 Structural Proteins

The amount of detected S protein in each inactivated vaccine was measured by the above method and the results are listed in Table 2. As shown in Table 2, the RSD of the detected S protein in three batches of COVID-19 (wild-type strain) inactivated vaccine samples from company B was 5.8%, showing a stable production progress of COVID-19 (wild-type strain) inactivated vaccines. However, the RSD of detected S protein in three batches of COVID-19 (Omicron strain) inactivated vaccine samples from the same company reached up to 32.9%, which is much higher than for the wild-type one. These results indicated that the production progress of the COVID-19 (wild-type strain) inactivated vaccine is more stable than vaccine made of inactivated COVID-19 (variant strain). It is worth noting that the results of the vaccine samples from company A could also support the conclusion mentioned above. In addition, the average of detectable S protein in three batches of COVID-19 (wild-type strain) inactivated vaccine samples from company B was 2.938 µg, but the value in COVID-19 (Omicron strain) inactivated vaccine samples was just 0.6888 µg. Similarly, COVID-19 (wild-type strain) inactivated vaccine samples from company A also showed a relatively higher S protein levels compared with Omicron strain inactivated vaccines. These results may be due to the production process of inactivated vaccines being more suitable for wild-type strain vaccine production.

In addition, the amount of detected S protein in each recombinant protein vaccine was also determined via this method. As shown in Table 3, the RSD of detected S protein in three batches of wild subtype recombinant protein vaccines was relatively low, which is comparable to the COVID-19 (wild-type strain) inactivated vaccine samples from company A or company B, revealing a stable S protein level between different batches. Nevertheless, compared with the inactivated vaccine, the recombinant protein vaccine seems to have more robust production technology, according to the stability of S protein expression in variant subtype vaccine from batch to batch (Table 2 and Table 3). This result may be caused by the nature of these two different vaccine production techniques. For inactivated virus vaccines, organisms like viruses need to be largely proliferated in the production process, which is easily susceptible to many uncertain factors for variant strain vaccines. However, the production process in recombinant protein vaccines aims to synthesize protein products simply at the molecular level, which may be relatively robust compared with inactivated virus vaccines.

**Figure 3:**
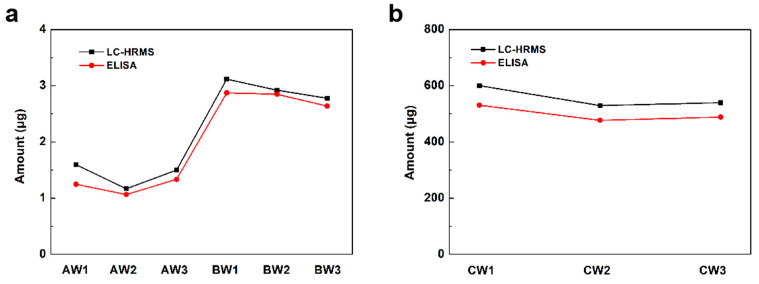
The amount of detected S protein in each (**a**) inactivated vaccine sample and (**b**) recombinant protein vaccine sample by LC-HRMS and ELISA.

To validate the quantification of this LC-HRMS-based method, a commercial S protein ELISA kit which could quantify wild subtype S proteins was used in this study. As shown in Figure 3, the results measured by LC-HRMS matched well with the ELISA results. The results confirmed the reliability of this LC-HRMS method for S protein quantitation in inactivated or recombinant vaccines.

### 3.3. Quantification of Non-COVID-19 Proteins

As an important type of non-COVID-19 protein in vaccine quality control, the content of total detected host cell proteins in each inactivated vaccine sample was measured using this method. As shown in Table 4, the average of total detected host cell proteins in three batches of COVID-19 (wild-type strain) inactivated vaccine samples from company B was 761.9 µg, but this value in the products from company A was just 137.9 µg. Analogously, the average content of total detected host cell proteins in three batches of COVID-19 (Omicron strain) inactivated vaccine samples from company B was also much higher than that from company A. These results showed a significant difference between company A and company B in HCP content. In addition, the total content and species number of detected host cell proteins in each recombinant protein vaccine were also measured by this method. As shown in Table 4, the average of total content and species number of detected host cell proteins in three batches of each subtype recombinant protein vaccine were obviously lower than that in inactivated vaccines. This may be due to it being easier to control the amount of HCP residue in the production process of recombinant protein vaccines.

As shown in Table 5, the relative proportion of total detected host cell proteins to S protein in each inactivated vaccine was very high, indicating that the amount of HCPs was far beyond the S protein. However, the relative proportion of total detected host cell proteins to S proteins in each recombinant protein vaccine was much lower than that of the inactivated vaccines (Table 5 and Table 6). This may depend on the principles of these two different types of vaccines. In recombinant protein vaccines, a target protein gene was integrated into a host cell genome to continuously produce the target protein at a large scale, which belongs to the scope of synthetic biology. However, inactivated vaccines aim to produce intact viruses through parasitic relations, which is a more complicated biological process than just protein production.

To date, the specific functions of the high content of HCPs in inactivated vaccine are not clear. Although inactivated vaccines contain high content of HCPs, it has not been conclusively demonstrated whether this is a defect of this type of vaccine. HCP residues may interfere with the immune response and induce toxic side effects. Further, HCP may also trigger protein–protein interactions to stimulate immune responses in a synergistic manner [17]. Therefore, it is hard to judge which type of vaccine has a better immune effect simply based on the content and species of HCPs. More studies need to be conducted to understand the specific effects of HCPs on immunological processes.

### 3.4. Comparing the Vaccines of Different Mutant of COVID-19

**Figure 4:**
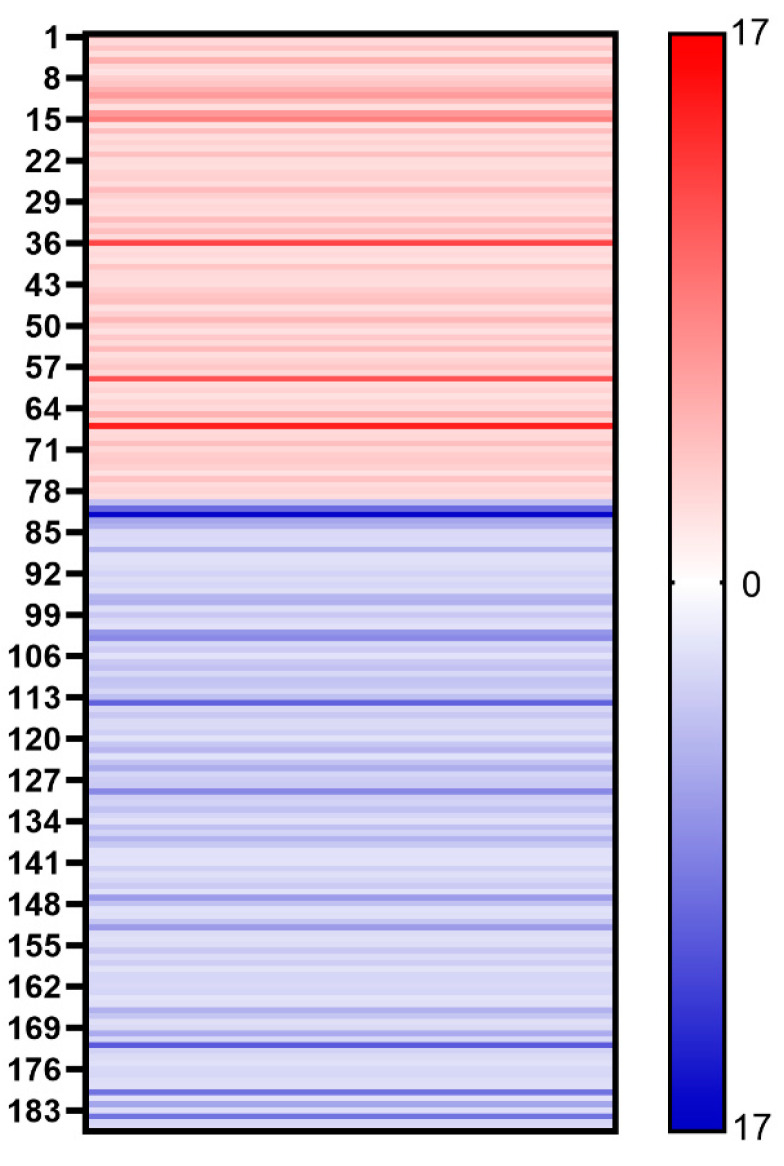
Heat map shows the changes of different host cell proteins. Red stripe represents the multiple of each up-regulated host cell protein in vaccine samples (AO/AW) and blue stripe represents the multiple of each down-regulated host cell protein in vaccine samples (AW/AO).

We further compared the protein content of different mutants of COVID-19. Despite the similar proteomes of the AW and AO vaccines, there are 79 host cell proteins whose abundance increased in the vaccine of the Omicron strain (Figure 4). On the other hand, the abundance of 107 host cell proteins was found to be decreased in vaccines of the Omicron strain. Gene Ontology (GO) analysis demonstrated the differences of the host cell proteomes. Up-regulated proteins in AO are enriched in cytoplasm (GO:0005737, 41 vs. 25), while down-regulated proteins are in the extracellular regions (GO:0005576, 4 vs. 11), lysosomes (GO:0005764, 1 vs. 7) and chromosomes (GO:0005694, 0 vs. 7). As for functions of differential regulated proteins, down-regulated proteins in AO are involved in ATP binding (GO:0005524) and hydrolase activity (GO:0016787), while up-regulated proteins are in RNA-binding (GO:0003723) and apoptotic processes (GO:0006915). These results suggest that the infection of the wild-type and Omicron strains may stimulate different responses of host cells [18], which may be used for optimizing production process, such as the MOI and medium for cell production.

**Figure 5:**
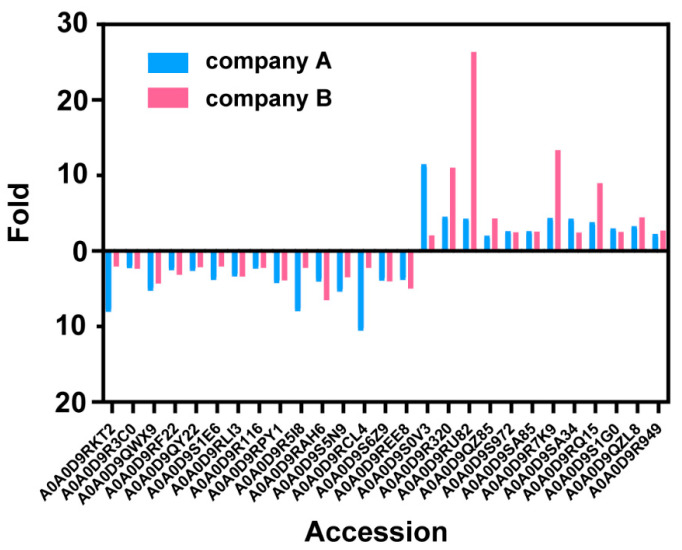
The changes of each down- or up-regulated host cell protein in two companies’ vaccine samples.

To find if the difference in proteomes is from the infection of different strains, we compared the proteomes of the host cells from another vaccine vendor (BW and BO). The core histones and apoptotic-related proteins are both up-regulated for both vendors, supporting that these differences are from the type of virus used (Figure 5). However, most regulated proteins are not the same, which suggests that the different production processes contribute to the host cell proteome.

## 4. Conclusions

In this work, we developed a nano LC-MS method for the simultaneous analysis of S proteins and HCP residues of COVID-19 inactivated vaccine products and recombinant vaccine products. By using this novel method, thousands of proteins, both known and unknown, can be qualitatively and quantitatively analyzed with only one injection. Based on this, the proteomes of 15 batches of COVID-19 inactivated vaccine samples from two companies and 12 batches of COVID-19 recombinant protein vaccine samples from one company were successfully analyzed, which provided a significant amount of valuable information. Samples in different batches or from different companies can be systematically contrasted in this way, offering powerful supplements for existing quality standards. In the future, this method can also be used for global monitoring during production (e.g., virus/host cell metabolism, MOI optimization, etc.), which may provide more supporting information for process improvement. This strategy paves the way for profiling proteomics in complex samples and provides a novel perspective on the quality evaluation of bio-macromolecular drugs.

## Figures and Tables

**Table 1:** The details of eluent gradient in this study.

t/min	Mobile Phase A/%	Mobile Phase B/%
0	95	5
5	90	10
47	65	35
54	55	45
55	10	90
60	10	90

**Table 2:** The amount of S protein in each inactivated vaccine sample (unit, µg).

Vaccine Sample.	Batch No. 1	Batch No. 2	Batch No. 3	Average	RSD
AW	1.595	1.166	1.498	1.420	15.8%
AG	0.6993	1.648	0.5262	0.9578	63.1%
AO	0.6078	1.388	0.9796	0.9918	39.3%
BW	3.116	2.920	2.778	2.938	5.8%
BO	0.9307	0.6546	0.4811	0.6888	32.9%

**Table 3:** The amount of S protein in each recombinant protein vaccine (unit, µg).

Vaccine Sample	Batch No. 1	Batch No. 2	Batch No. 3	Average	RSD
CW	600.2	529.6	539.9	556.6	6.9%
CB	509.1	443.7	500.3	484.4	7.3%
CD	470.9	468.8	488.4	476.0	2.3%
CO	247.4	253.6	254.6	251.9	1.5%

**Table 4:** The total content and species number of detected host cell proteins in each vaccine sample.

	Total Content (Unit, µg)				Species Number			
Vaccine Sample	Batch No. 1	Batch No. 2	Batch No. 3	Average	Batch No. 1	Batch No. 2	Batch No. 3	Average
AW	141.3	162.7	109.7	137.9	3571	3633	3623	3609
AG	86.57	25.20	60.84	57.54	2986	3007	3041	3011
AO	94.70	118.7	88.26	100.6	3575	3598	3543	3572
BW	687.0	1085	513.6	761.9	4105	4098	4090	4098
BO	1306	490.1	932.9	909.7	4128	4123	4122	4124
CW	45.67	47.47	53.01	48.72	240	344	504	363
CB	55.40	70.97	27.48	51.28	465	247	236	316
CD	28.38	43.41	80.69	50.83	258	282	527	356
CO	61.62	56.16	45.75	54.51	326	304	237	289

**Table 5:** The relative proportion of total detected host cell proteins to S protein in each inactivated vaccine (unit, µg).

Vaccine Sample	AW	AG	AO	BW	BO
HCP	137.9	57.54	100.6	761.9	909.7
S protein	1.420	0.9578	0.9918	2.938	0.6888
Ratio	97	60	101	259	1321

**Table 6:** The relative proportion of total detected host cell proteins to S protein in each recombinant protein vaccine (unit, µg).

Vaccine Sample	CW	CB	CD	CO
HCP	48.72	51.28	50.83	54.51
S protein	556.6	484.4	476.0	251.9
Ratio	0.08753	0.1059	0.1068	0.2164

## Data Availability

Sample raw MS files are available upon request.
